# Pharmacokinetic Evaluation of Risperidone and Its Active Metabolite When Risperidone Oral Solution Is Mixed with Black Tea in Rats

**DOI:** 10.3390/ph19060855

**Published:** 2026-05-29

**Authors:** Yosuke Nishikawa, Hiroyuki Suzuki, Ryusuke Ouchi, Taisuke Konno, Kensuke Usui, Takashi Watanabe, Kouji Okada, Shigeki Kisara, Yuriko Murai

**Affiliations:** 1 Clinical Pharmacy Practice Center, Faculty of Pharmaceutical Sciences, Tohoku Medical and Pharmaceutical University, 4-4-1 Komatsushima, Aoba-ku, Sendai 981-8558, Japan; kisara.shigeki@tohoku-mpu.ac.jp; 2 Department of Pharmacy, Tohoku Medical and Pharmaceutical University Hospital, 1-12-1 Fukumuro, Miyagino-ku, Sendai 983-8512, Japan; rouchi@tohoku-mpu.ac.jp (R.O.); tkonno@tohoku-mpu.ac.jp (T.K.); k-usui@tohoku-mpu.ac.jp (K.U.); watanabe.taka@tohoku-mpu.ac.jp (T.W.); kokada@tohoku-mpu.ac.jp (K.O.); 3 Division of Clinical Pharmaceutics, Faculty of Pharmaceutical Sciences, Tohoku Medical and Pharmaceutical University, 4-4-1 Komatsushima, Aoba-ku, Sendai 981-8558, Japan; y-murai@tohoku-mpu.ac.jp; 4 Division of Clinical Pharmaceutics and Pharmacy Practice, Faculty of Pharmaceutical Sciences, Tohoku Medical and Pharmaceutical University, 1-12-1 Fukumuro, Miyagino-ku, Sendai 983-8512, Japan; 5 Division of Clinical Pharmacotherapeutics, Faculty of Pharmaceutical Sciences, Tohoku Medical and Pharmaceutical University, 4-4-1 Komatsushima, Aoba-ku, Sendai 981-8558, Japan; 6 Division of Community Medicine and Pharmacy, Faculty of Pharmaceutical Sciences, Tohoku Medical and Pharmaceutical University, 4-4-1 Komatsushima, Aoba-ku, Sendai 981-8558, Japan

**Keywords:** risperidone, black tea, interactions, pharmacokinetic

## Abstract

**Background/Objectives**: Risperidone oral solution (RIS-OS) is an easy-to-administer treatment for schizophrenia designed to improve medication adherence and rapid onset of effect. Mixing RIS-OS with beverages such as black tea is prohibited due to reduced RIS concentrations observed in vitro, despite the absence of pharmacokinetic data. In this study, we evaluated the pharmacokinetics of RIS and its active metabolite 9-OH-RIS in rats following oral administration with black tea. **Methods**: Male Wistar rats received RIS-OS intravenously or orally as a water or Dimbula black tea mixture; serial tail-vein blood samples were collected as dried blood spots, RIS and 9-OH-RIS were quantified using HPLC/ESI-MS/MS, and pharmacokinetic parameters were calculated and compared using Welch’s *t*-test. **Results**: Compared with the water mixture, the black tea mixture significantly reduced RIS C_max_, while T_max_ and AUC remained unchanged. Furthermore, AUMC and MRT were significantly higher. The results were similar for 9-OH-RIS. Despite reduced RIS content in vitro, no difference in absolute bioavailability was observed in vivo. Although only one black tea variety was tested, evaluating additional varieties may help identify the components responsible for reducing RIS content. **Conclusions**: Mixing RIS-OS with black tea may delay absorption without reducing overall exposure, providing evidence that may contribute to safer guidance regarding beverage coadministration.

## 1. Introduction

While cases of patients discontinuing medication have been reported in the pharmacological treatment of schizophrenia, discontinuation of antipsychotic drugs may lead to the recurrence or worsening of psychotic symptoms. Thus, ensuring medication adherence is considered one of the key support measures [1]. Risperidone (RIS) is a standard second-generation antipsychotic used to treat patients, including those with first-episode schizophrenia. It is available in various formulations, including oral tablets and long-acting injectables [2]. In particular, RIS oral solution (RIS-OS) is useful for patients who have difficulty adhering to medication, such as those in acute agitated states or those who feign non-compliance.

While RIS-OS is convenient to use, its administration is restricted. RISPERDAL^®^ product information explicitly states that concomitant use with cola beverages and black tea is contraindicated [3]. While mixing with tea extracts reduces RIS content in vitro, accompanied by complex formation with tea components such as catechins [4,5], the specific tea components responsible, as well as whether they are dose- or concentration-dependent, have not been clarified. Furthermore, no studies have explored the impact of such combinations on pharmacokinetics or the onset of therapeutic effects. However, regarding the clinical use of RIS-OS, a previous study reported that approximately one-fifth of outpatients took the medication mixed with beverages, and some patients mixed it with tea or cola [6]. Although these results are limited to a specific region, the characteristics of the target diseases and the fact that black tea production and consumption are increasing globally year by year [7] suggest that medications may plausibly be mixed with black tea to improve ease of administration.

RIS is a weakly basic drug with a pK_a_ of 8.24 [3]. Furthermore, RIS-OS’s formulation is adjusted to acidic conditions to maintain the RIS. Given these physicochemical properties, the dissolution behavior and absorption process of RIS-OS may be altered by gastrointestinal tract or interactions with concomitant substances. Therefore, we considered that pharmacokinetic verification through in vivo studies is essential for understanding the fundamental aspects of the drug’s efficacy and safety. With the aim of obtaining clinically useful and reliable pharmacokinetic information on RIS-OS, we administered a mixture of RIS-OS and black tea to rats. To evaluate the effects on RIS absorption and metabolism, total concentrations were measured and pharmacokinetic parameters were calculated.

## 2. Results

### 2.1. Validation of Analytical Method for RIS and 9-OH-RIS Concentrations in Rat Whole Blood

To minimize analytical variability during extraction and ionization, d4-RIS was used as an internal standard (IS). Quantification of RIS and 9-OH-RIS was performed using an IS method based on the peak area ratios of each analyte to the IS, and calibration curves were constructed accordingly. The calibration curves for RIS and 9-OH-RIS showed good linearity over the concentration range of 1–1000 ng∙mL^−1^, with coefficients of determination (*R*^2^) greater than 0.999 for both analytes. For RIS, recovery and accuracy (relative error, RE%) ranged from −10.1 to 0.594%, while precision (coefficient of variation, CV%) ranged from 1.95 to 9.24%. For 9-OH-RIS, RE ranged from −9.95 to 7.45%, and CV ranged from 2.53 to 7.31%. These results suggest that the analytical method provided adequate accuracy and precision for quantifying RIS and 9-OH-RIS.

### 2.2. Area Under the Whole-Blood Concentration–Time Curve of Intravenous (AUC_i.v.0–∞_)

To calculate AUC_i.v._, RIS-OS was administered as a single intravenous dose via the caudal vein in the intravenous injection group (*n* = 3). The resulting whole-blood concentration–time curve was assumed to follow a one-compartment model with first-order elimination. The elimination rate constant (k_el_) was calculated by log-linear regression of the terminal phase, and the whole-blood concentration immediately after administration (C_0_) was determined by extrapolation using the regression line. The mean AUC_i.v._ was 15,851 ng·min∙mL^−1^.

### 2.3. Whole-Blood RIS Concentration Following Oral Administration

**Figure 1:**
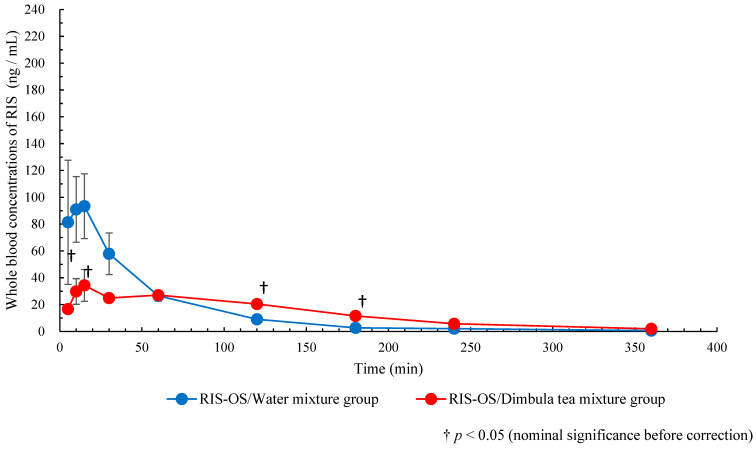
Whole-blood concentration–time profiles of RIS in the RIS-OS/water-mixture and RIS-OS/Dimbula tea-mixture groups (*n* = 3).

**Figure 2:**
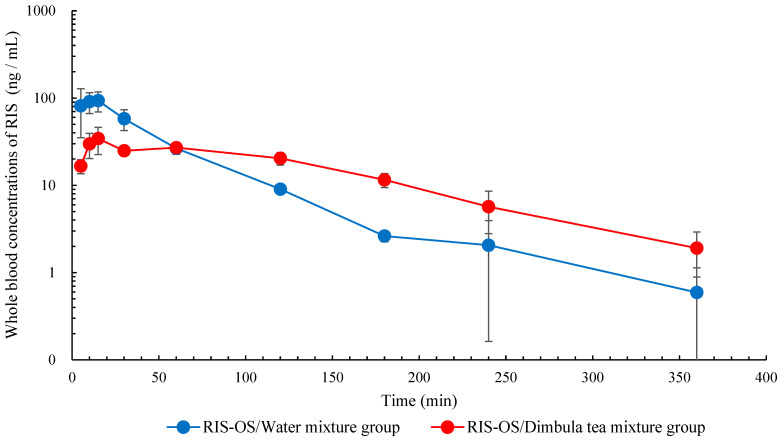
Semi-logarithmic plot of the whole-blood concentration–time profiles of RIS in the RIS-OS/water-mixture and RIS-OS/Dimbula tea-mixture groups (*n* = 3).

Whole-blood concentration–time profiles of RIS in the RIS-OS/water-mixture and RIS-OS/Dimbula tea-mixture groups are shown in Figure 1 and Figure 2. The mean C_max_ was 113 ng∙mL^−1^ in the RIS-OS/water-mixture group and 37.6 ng∙mL^−1^ in the RIS-OS/Dimbula tea-mixture group, with the RIS-OS/Dimbula tea-mixture group showing a significantly lower value. The mean T_max_ was 10.0 min for the RIS-OS/water-mixture group and 9.0 min for the RIS-OS/Dimbula tea-mixture group, with no significant difference between the two groups.

Table 1 shows the pharmacokinetic parameters of RIS in the RIS-OS/water-mixture and RIS-OS/Dimbula tea-mixture groups. The mean AUC was 5318 ng·min∙mL^−1^ in the RIS-OS/water-mixture group and 5160 ng·min∙mL^−1^ in the RIS-OS/Dimbula tea-mixture group, with no significant difference between the two groups. The mean area under the (first) moment curve (AUMC) was 3.2 × 10^5^ ng·min^2^∙mL^−1^ in the RIS-OS/water-mixture group and 6.3 × 10^5^ ng·min^2^∙mL^−1^ in the RIS-OS/Dimbula tea-mixture group. Additionally, the mean residence time (MRT) was 60 min for the RIS-OS/water-mixture group and 121 min for the RIS-OS/Dimbula tea-mixture group, with the RIS-OS/Dimbula tea-mixture group showing significantly higher values in both parameters. The mean k_a_ was 0.251 min^−1^ for the RIS-OS/water-mixture group and 0.162 min^−1^ for the RIS-OS/Dimbula tea-mixture group, with no significant difference between the two groups. The mean k_el_ was 0.00857 min^−1^ for the RIS-OS/water-mixture group and 0.00987 min^−1^ for the RIS-OS/Dimbula tea-mixture group, with no significant difference between the two groups. The mean t_1/2_ was 80.8 min for the RIS-OS/water-mixture group and 70.2 min for the RIS-OS/Dimbula tea-mixture group, with no significant difference between the two groups.

Compared with the RIS-OS/water-mixture group, the RIS-OS/Dimbula tea-mixture group showed a significantly lower C_max_, whereas T_max_ did not differ significantly between the groups. Blood concentrations were reversed 60 min after administration, and no significant difference was observed in AUC. In contrast, AUMC and MRT were significantly higher in the RIS-OS/Dimbula tea-mixture group.

### 2.4. Whole-Blood 9-OH-RIS Concentration Following Oral Administration

**Figure 3:**
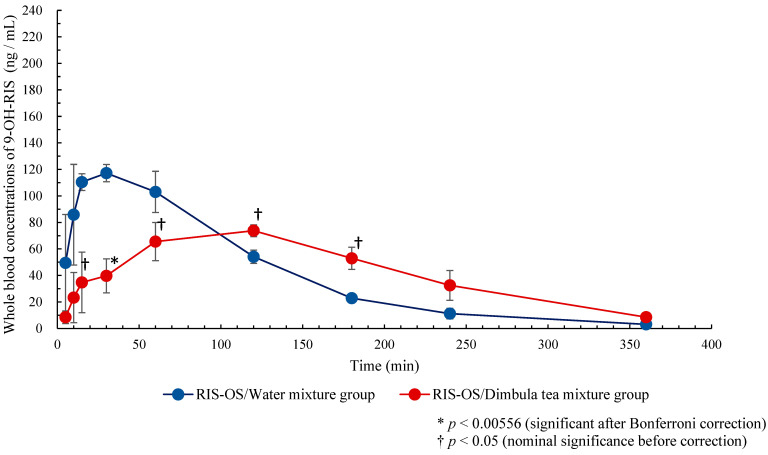
Whole-blood concentration–time profiles of 9-OH-RIS in the RIS-OS/water-mixture and RIS-OS/Dimbula tea-mixture groups (*n* = 3).

**Figure 4:**
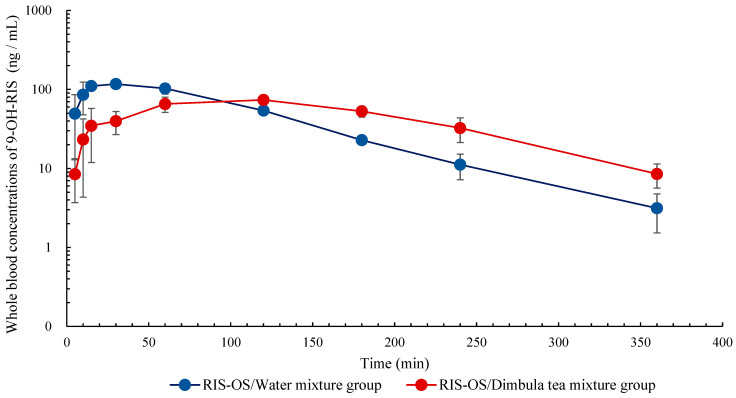
Semi-logarithmic plot of the whole-blood concentration–time profiles of 9-OH-RIS in the RIS-OS/water-mixture and RIS-OS/Dimbula tea-mixture groups (*n* = 3).

Whole-blood concentration–time profiles of 9-OH-RIS in the RIS-OS/water-mixture and RIS-OS/Dimbula tea-mixture groups are shown in Figure 3 and Figure 4. The mean C_max_ was 119.2 ng∙mL^−1^ in the RIS-OS/water-mixture group and 74.7 ng∙mL^−1^ in the RIS-OS/Dimbula tea-mixture group, with the RIS-OS/Dimbula tea-mixture group showing a significantly lower value. The mean T_max_ was 35 min for the RIS-OS/water-mixture group and 80 min for the RIS-OS/Dimbula tea-mixture group, with no significant difference between the two groups. Whole-blood concentrations in the RIS-OS/Dimbula tea-mixture group were significantly lower at the 30 min time point.

Table 2 shows the pharmacokinetic parameters of 9-OH-RIS in the RIS-OS/water-mixture and RIS-OS/Dimbula tea-mixture groups. The mean AUC was 15,100 ng·min∙mL^−1^ in the RIS-OS/water-mixture group and 16,223 ng·min∙mL^−1^ in the RIS-OS/Dimbula tea-mixture group, with no significant difference between the two groups. The mean AUMC was 1.33 × 10^6^ ng·min^2^∙mL^−1^ for the RIS-OS/water-mixture group and 2.2 × 10^6^ ng·min^2^∙mL^−1^ in the RIS-OS/Dimbula tea-mixture group. Additionally, the mean MRT was 87.5 min in the RIS-OS/water-mixture group and 136 min in the RIS-OS/Dimbula tea-mixture group, with significantly higher values observed in the RIS-OS/Dimbula tea-mixture group. The mean k_el_ was 0.01096 min^−1^ for the RIS-OS/water-mixture group and 0.01029 min^−1^ for the RIS-OS/Dimbula tea-mixture group, with no significant difference between the two groups. The mean t_1/2_ was 63.2 min in the RIS-OS/water-mixture group and 67.4 min in the RIS-OS/Dimbula tea-mixture group, with no significant difference between the two groups.

Compared with the RIS-OS/water-mixture group, the RIS-OS/Dimbula tea-mixture group showed a significantly lower C_max_, whereas T_max_ remained unchanged. A reversal in total concentration occurred 120 min after administration, while AUC remained unchanged. Furthermore, AUMC and MRT were significantly higher in the RIS-OS/Dimbula tea-mixture group.

The metabolic ratio did not significantly differ between the two groups.

### 2.5. Whole-Blood Active Moiety Concentration Following Oral Administration

**Figure 5:**
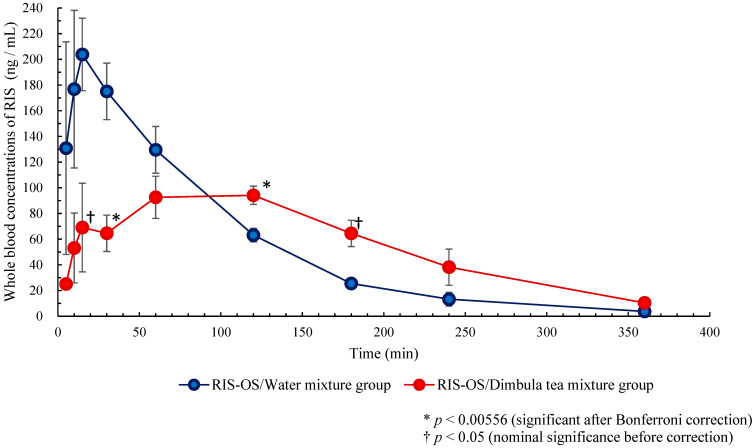
Whole-blood concentration–time profiles of the active moiety in the RIS-OS/water-mixture and RIS-OS/Dimbula tea-mixture groups (*n* = 3).

**Figure 6:**
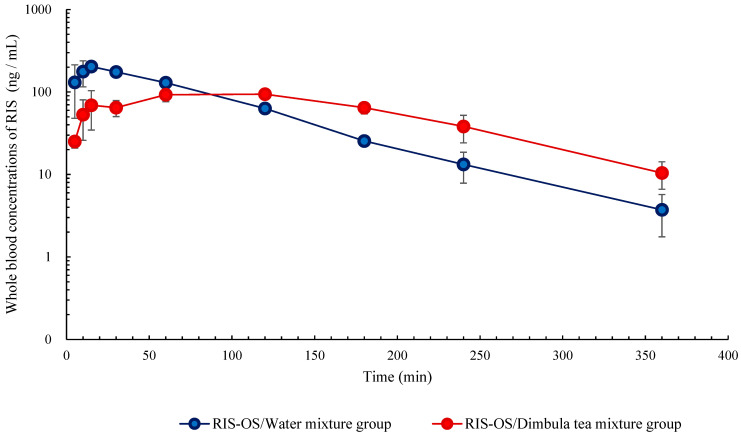
Semi-logarithmic plot of the whole-blood concentration–time profiles of the active moiety in the RIS-OS/water-mixture and RIS-OS/Dimbula tea-mixture groups (*n* = 3).

The combined concentration of RIS and 9-OH-RIS was defined as the active moiety. Whole-blood concentration–time profiles of the active moiety in the RIS-OS/water-mixture and RIS-OS/Dimbula tea-mixture groups are shown in Figure 5 and Figure 6. The mean C_max_ was 219 ng∙mL^−1^ in the RIS-OS/water-mixture group and 102.5 ng∙mL^−1^ in the RIS-OS/Dimbula tea-mixture group, with the RIS-OS/Dimbula tea-mixture group showing a significantly lower value. The mean T_max_ was 13 min for the RIS-OS/water-mixture group and 65 min for the RIS-OS/Dimbula tea-mixture group, with no significant difference between the two groups. Whole-blood concentrations in the RIS-OS/Dimbula tea-mixture group were significantly lower at 30 and 120 min.

Table 3 shows the pharmacokinetic parameters of the active moiety in the RIS-OS/water-mixture and RIS-OS/Dimbula tea-mixture groups. The mean AUC was 20,430 ng·min∙mL^−1^ in the RIS-OS/water-mixture group and 21,293 ng·min∙mL^−1^ in the RIS-OS/Dimbula tea-mixture group, with no significant difference between the two groups.

## 3. Discussion

RIS is primarily metabolized by CYP2D6 to its active metabolite, 9-hydroxyrisperidone (9-OH-RIS), in humans, and RIS and 9-OH-RIS exhibit similar affinities for dopamine D_2_ and serotonin 5-HT_2_ receptors [8]. Therefore, even when CYP2D6-mediated metabolism is altered, the impact on the active moiety (RIS + 9-OH-RIS) may be relatively limited [9]. In contrast, physicochemical interactions that reduce the amount of available RIS may directly decrease overall active moiety exposure. Although in vitro studies have shown that mixing RIS-OS with black tea reduces RIS content, to the best of our knowledge, no in vivo studies using such a mixture have been conducted to date. This study is the first to investigate the pharmacokinetics of RIS absorption and metabolism following oral administration of a mixture of RIS-OS and Dimbula tea in rats. The results show that, for both RIS and 9-OH-RIS, the Dimbula tea-mixture group exhibited no significant difference in AUC compared with the water-mixture group; however, AUMC and MRT were significantly prolonged. Specifically, it was newly established that oral administration of RIS-OS mixed with Dimbula tea prolongs the absorption process.

Several in vitro studies have been conducted to investigate the mechanism by which RIS content decreases when it is mixed with black tea [5,10,11,12]. RIS is believed to form complexes with catechins and other compounds in tea leaf extracts. Dimbula tea used in this study is a distinct variety of Ceylon black tea grown in the highlands of Sri Lanka. While the polyphenol composition of black tea generally varies depending on its geographical origin and specific processing methods, Dimbula tea exhibits a unique chemical profile. Compared to other world-famous teas, such as Indian Darjeeling and various Chinese teas, Dimbula tea is characterized by a lower content of catechins and a significantly higher content of theaflavins [13]. Among the black teas produced in the high-altitude regions of Sri Lanka, Dimbula tea possesses a particularly high concentration of gallated theaflavins [14]. Furthermore, recent studies have reported that caffeine inhibits this complex formation [15]. While elucidating the components and mechanisms underlying such interactions is important, given the actual administration practices of RIS-OS, examining the impact of these interactions under conditions simulating real-world clinical use is essential. In the present study, administration of the mixed solution in rats did not result in reduced absolute RIS bioavailability; therefore, these findings demonstrate the value of conducting further studies that more closely mimic clinical conditions.

Pharmacokinetic analyses in this study were performed using whole-blood concentrations rather than plasma concentrations. Therefore, direct comparison with previous plasma-based studies should be interpreted cautiously. Previous reports have shown blood-to-plasma ratios of approximately 0.67 in humans and 0.78 in rats for RIS [16], suggesting matrix-dependent differences in measured concentrations and pharmacokinetic parameters.

Regarding total RIS concentrations, the fact that no significant difference in AUC was observed in the Dimbula tea-mixture group indicates that the extent of absorption was maintained. These findings suggest that RIS in the complex formed with black tea components redissolved within the rat’s body. In fact, the partition coefficient of RIS in 1-octanol/citric-phosphate buffer (pH 2.2) is reportedly 1.28 × 10^−24^, indicating that the hydrophilicity of RIS increases under acidic conditions. Furthermore, previous studies have suggested that the piperidine nitrogen atom of RIS may be involved in complex formation with tea leaf catechins [11,12], and this nitrogen atom is thought to determine the basicity of RIS (pK_a_ 8.24). We confirmed that, after mixing RIS oral solution with Dimbula tea and centrifugation, the resulting precipitate could be redissolved in JP 1st fluid (pH 1.2; 2.0 g sodium chloride and 7.0 mL diluted hydrochloric acid in 1000 mL purified water). Under these conditions, the amount of RIS recovered from the precipitate was approximately equal to the decrease observed in the supernatant, and the combined amount in both fractions was close to the theoretical value. These findings may support the possibility that RIS precipitation induced by Dimbula tea is at least partially reversible under acidic conditions. Therefore, in acidic environments such as gastric juice, protonation of this nitrogen atom may reduce the stability of the complex and promote its dissociation. Consequently, redissolution of RIS is likely to be promoted. Based on the above, the results of the current study suggest that the complex formed between RIS and black tea components may exhibit pH-dependent reversible behavior. Such pH-dependent complex formation and dissociation may influence the dissolution behavior and absorption process of RIS within the gastrointestinal tract. In particular, RIS, whose solubility was temporarily reduced due to complex formation with black tea components, may redissolve in the acidic environment of the stomach, thereby affecting pharmacokinetic parameters.

Furthermore, in the Dimbula tea-mixture group, C_max_ was significantly lower, whereas AUMC and MRT were significantly higher, with no significant differences observed in T_max_, k_a_, or k_el_. The unchanged k_el_ was not unexpected, as the same formulation was used regardless of the mixture. In other words, the prolongation of the residence time in the body was likely primarily due to the absorption process rather than the elimination process. The lack of a significant difference in k_a_ could be due to the dissociation of the complex within the gastrointestinal tract via the aforementioned mechanism, resulting in the absorption process being divided into multiple stages. Specifically, while a portion of RIS remaining in solution continues to be rapidly absorbed, the fraction of RIS forming complexes undergoes delayed absorption due to the need for redissolution. As a result, the absorption peak was flattened, increasing its contribution to concentrations in the late phase [17]. Although previous studies in patients in the chronic phase reported no correlation between treatment response and plasma concentrations of RIS and 9-OH-RIS [18], the present results showed a decrease in C_max_ and a prolongation of MRT, suggesting that the drug’s efficacy may either be reduced or the duration of its effects may be prolonged.

Regarding total 9-OH-RIS concentrations, similar to RIS, C_max_ was significantly lower, whereas AUMC and MRT were significantly higher in the Dimbula tea group compared with the water group. However, no significant differences were observed in T_max_ or k_el_. Previous reports have indicated that theaflavins in black tea may inhibit OATP2B1 [19], suggesting a potential effect of black tea alone on drug absorption and metabolism. However, no significant difference in metabolic ratio was observed between the groups, and because RIS is a basic drug and is not a substrate for OATP, black tea itself likely did not affect RIS absorption or metabolism. Therefore, the results for 9-OH-RIS are consistent with the view that the observed changes reflect the aforementioned changes in RIS absorption.

The limitations of this study include differences in drug-metabolizing enzymes and the gastrointestinal environment between rats and humans, which limit direct extrapolation of the findings to humans; the use of a single type of black tea, meaning the results may not be generalizable to black tea in general; and the spiked DBS samples lacked short-term and long-term stability evaluations, and further validation is required to confirm sample stability under various storage conditions. In addition, this study was an exploratory animal experiment, and the sample size for each group was relatively small (*n* = 3), resulting in limited statistical power. Therefore, results showing no significant differences in certain pharmacokinetic parameters, including T_max_, should be interpreted with caution, taking into account the possibility of a type II error.

This study was conducted as an exploratory preclinical pharmacokinetic study. Further verification through dedicated in vitro dissolution and pH-shift studies designed for rats and humans is required to elucidate the detailed mechanisms underlying the pH-dependent solubility of the complex. The delay in absorption rate due to multistage absorption observed in the present study may apply to other types of tea-based beverages as well. Specifically, because black tea beverages other than green and Dimbula tea caused a smaller decrease in RIS content in vitro than Dimbula tea, these teas may not alter AUC but would result in higher C_max_ values in vivo. Expanding the range of tea-based beverages studied and conducting additional pharmacokinetic and pharmacodynamic studies would be valuable. This study highlights that, when evaluating drug interactions, clinical practice should assess not only drug stability but also pharmacokinetic effects under conditions in which interactions occur during administration.

## 4. Materials and Methods

### 4.1. Animals

Male Wistar rats (8 weeks old, approximately 260 g; CLEA Japan, Inc., Tokyo, Japan) were used in this study. The rats were acclimatized under controlled environmental conditions (temperature, 23.0 ± 1 °C; humidity, 55 ± 7%) with a 12 h light/dark cycle (8:00 to 20:00), and had free access to food and water before fasting overnight (approximately 12 h) prior to administration, while water remained available. Three rats were assigned to each of the following groups: intravenous injection, oral administration with water (RIS-OS/water mixture), and oral administration with tea (RIS-OS/Dimbula tea mixture).

### 4.2. Materials

RIS-OS (RISPERDAL^Ⓡ^ Oral Solution 1 mg∙mL^−1^) was purchased from Janssen Pharmaceutical K.K. (Tokyo, Japan). Acetonitrile, methanol, ultra-pure water, ammonium acetate, and formic acid used for liquid chromatography-mass spectrometry were obtained from FUJIFILM Wako Pure Chemical Co. (Osaka, Japan). RIS, 9-OH-RIS, and d_4_-RIS were purchased from Toronto Research Chemicals (Toronto, ON, Canada). Tap water was used to prepare the oral solution. Dimbula tea was prepared from commercially available tea bag products (Karel Capek Company, Tokyo, Japan).

### 4.3. Drug Administration

Drug administration was performed under open-drop anesthesia using inhaled isoflurane. Intravenous injection was performed using a restraint device, whereas oral administration was performed under manual handling. The dose of RISPERDAL^®^ oral solution (1 mg mL^−1^) was 1.25 mg kg^−1^ for both intravenous injection and oral administration. For oral administration with water or tea, the solution’s concentration was adjusted to approximately 0.091 mg mL^−1^.

### 4.4. Sample Collection

**Figure 7:**
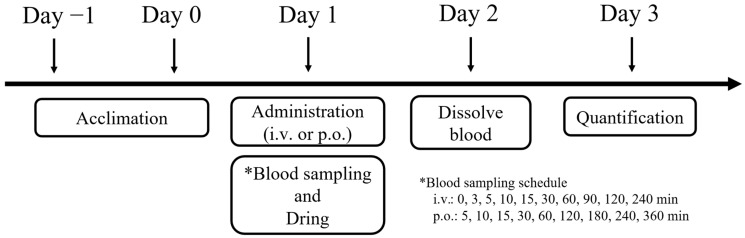
Experiment schedule.

Blood samples were collected from the tail vein after intravenous injection or oral administration of RIS-OS. Dried-blood-spot (DBS) sampling was employed to minimize blood collection volume and reduce the number of rats used. Blood samples (100 μL) were applied to blood-sampling paper (ADVSNTEC Toyo Co., Ltd., Tokyo, Japan). Blood-sampling times after intravenous injection and oral administration were 0, 3, 5, 10, 15, 30, 60, 90, 120, and 240 min and 5, 10, 15, 30, 60, 120, 180, 240, an 360 min, respectively (Figure 7). DBS samples were dried at room temperature for 12 h and analyzed within 1 d after preparation. Short- and long-term stability studies of spiked DBS samples were not performed in the present study.

### 4.5. Quantification of RIS and 9-OH-RIS Concentrations in Rat Whole Blood

Each sample was air-dried overnight under controlled laboratory conditions (temperature, 25 ± 1 °C; relative humidity, 42 ± 13%). For extraction, 200 μL of water was added to each sample and allowed to stand for 5 min, followed by vortex mixing for 1 min. Subsequently, 800 µL of 0.1% HCOOH/MeOH was added, and the mixture was vortex-mixed for 5 min and centrifuged at 10,000 rpm for 5 min. The supernatant was filtered through a 0.2 µm membrane filter and used for quantification. Quantitative analysis was conducted using HPLC/ESI-MS/MS with d_4_-RIS as an IS.

HPLC was performed using a Nexera XR system (Shimadzu, Kyoto, Japan) equipped with an HPLC system controller (CBM-20A), fluid delivery unit (LC-30AD), autosampler (SIL-30AC), column oven (CTO-20A), and switching valve (FCV-20AH2). Separation was achieved using a CAPCELL PAK C18 analytical column (2.0 mm i.d. × 100 mm, 3 μm; Shimadzu) with an MGIII cartridge (C18, 3 μm, 2.0 mm i.d. × 10 mm, Shimadzu), maintained at 40 °C. LC/ESI-MS/MS was conducted using an LCMS-8040 triple-quadrupole mass spectrometer (Shimadzu) equipped with an electrospray ionization (ESI) probe. The nebulizer gas flow rate was set to 3 L min^−1^, the desolvation line temperature to 250 °C, and the heat block temperature to 400 °C. Data acquisition and processing were performed using LabSolution software 5.86 (Shimadzu).

Ionization of RIS and 9-OH-RIS was verified in the ESI mode. RIS exhibited a precursor ion at *m*/*z* 411.20 and a product ion at *m*/*z* 191.10. 9-OH-RIS exhibited a precursor ion at *m*/*z* 427.15 and a product ion at *m*/*z* 207.10. The IS (d_4_-Risperidone) exhibited a precursor ion at *m*/*z* 415.25 and a product ion at *m*/*z* 195.10. The collision energy producing the strongest product ion signal was 30 V. The mobile phase consisted of solution A (20 mM ammonium acetate in water) and solution B (methanol), delivered isocratically at a ratio of A:B = 50:50 with a flow rate of 0.2 mL min^−1^ (1.01–13.0 min).

To remove impurities from whole-blood samples, a column-switching method using a trap column was applied. The trap column was an MAY-I ODS column (2.0 mm × 10 mm; Shimadzu). The mobile phase consisted of solution A (20 mM ammonium acetate in water) and solution B (methanol), delivered isocratically at a ratio of A:B = 95:5 with a flow rate of 0.5 mL min^−1^ (0.01–1 min, 13.1–15.0 min).

Peak specificity for RIS and 9-OH-RIS was evaluated. The retention times observed for the mixed sample were 11.6 min for RIS and 7.71 min for 9-OH-RIS, consistent with those obtained from individual injections of each compound.

The calibration range was set at 1–1000 ng/mL for RIS and 9-OH-RIS to cover a broad range of possible concentrations in rat DBS samples, including high concentrations at early sampling points after administration. This range was also selected to minimize the need for dilution and reanalysis of DBS samples, for which only a limited blood volume was available. The same QC concentration levels were used for RIS and 9-OH-RIS because both analytes were evaluated within the same analytical concentration range.

QC samples were prepared at 8.00, 80.0, and 800 ng/mL as low-, intermediate-, and high-QC samples, respectively.

### 4.6. Standard Solutions

Analytical standard solutions containing RIS and 9-OH-RIS were prepared by dilution with MeOH to final concentrations of 0, 1, 3, 10, 30, 100, 300, and 1000 ng∙mL^−1^. These solutions were used to generate standard curves, with d_4_-RIS as the IS.

### 4.7. Calculation of Pharmacokinetic Parameters

The area under the whole-blood concentration–time curve following intravenous administration (AUC_i.v.0–∞_) was calculated assuming a one-compartment model with first-order elimination using Equation (1), where C_0_ represents the extrapolated whole concentration at time zero. Time to reach the maximum concentration (T_max_) and the maximum whole-blood concentration (C_max_) were obtained from the plotted concentration–time curves. AUC and AUMC were calculated using the trapezoidal method. k_a_ was calculated using the nonlinear least-squares method in MULTI (Dr. Kazuhiko Yamaoka, Dr. Tatsuji Nakagawa, and Dr. Tsutomu Uno, National Institute of Hygienic Sciences, Japan) [Equation (2)]. k_el_ was calculated from the slope of the regression line obtained by the least-squares method during the elimination phase of the log concentration–time curve. Metabolic ratios were calculated as the ratio of the AUC of 9-OH-RIS to that of RIS using Equation (3). Other pharmacokinetic parameters were calculated using Equations (4)–(6).

(1) $$AUC_{i.v.0\text{–}\infty }=\frac{C_{0}}{k_{el}}$$

(2) $$c=\frac{F\cdot D\cdot k_{a}}{V_{d}\cdot \left(k_{a}-k_{el}\right)}\cdot \left(e^{-k_{el}\cdot t}-e^{-k_{a}\cdot t}\right)$$

(3) $$Metabolic\;ratio=\frac{AUC\text{\_}9\text{-}\mathrm{OH}\text{-}\mathrm{RIS}}{AUC\text{\_}\mathrm{RIS}}$$

(4) $$MRT=\frac{AUMC}{AUC}$$

(5) $$\frac{CL}{F}=\frac{D}{AUC}$$

(6) $$t_{1/2}=\frac{ln2}{k_{el}}$$

### 4.8. Statistical Analysis

Data are presented as the mean ± standard deviation. Differences were evaluated using Welch’s *t*-test in EZR 1.6 (Jichi Medical University, Saitama Medical Center, Saitama, Japan). Statistical significance was considered at *p* < 0.05.

## 5. Conclusions

Although in vitro studies have shown that mixing RIS-OS with black tea reduces RIS content, in vivo studies in rats demonstrated that, compared with mixing with water, the absolute bioavailability of RIS in RIS-OS mixed with black tea remained unchanged, whereas MRT was significantly prolonged due to a multi-step absorption process. Similar findings were observed for 9-OH-RIS.

Based solely on previous in vitro data, concerns focused on the possibility that mixing RIS-OS with black tea could weaken or eliminate its effects. However, the present in vivo study suggests that such coadministration may instead delay the onset of action and prolong the duration of effect. Expanding the range of tea-based beverages examined and conducting pharmacodynamic studies may contribute to generating clinically useful and reliable pharmaceutical information regarding RIS-OS.

## Figures and Tables

**Table 1:** Pharmacokinetic parameters of RIS following oral administration for the RIS-OS/water-mixture group and the RIS-OS/Dimbula tea-mixture group.

Parameter	RIS-OS/Water-Mixture Group	RIS-OS/Dimbula Tea-Mixture Group
T_max_ (min)	10.0 ± 5.1	9.0 ± 6.7
C_max_ (ng mL^−1^)	113 ± 19	37.6 ± 9.9 **
AUC (ng min mL^−1^)	5318 ± 247	5160 ± 410
AUMC (ng min^2^ mL^−1^)	3.2 × 10^5^ ± 8.2 × 10^4^	6.3 × 10^5^ ± 1.3 × 10^5^ *
MRT (min)	60 ± 17	121 ± 17 *
CL/F (mL min kg^−1^)	61.3 ± 2.9	64.4 ± 5.3
k_a_ (min^−1^)	0.251 ± 0.097	0.162 ± 0.040
k_el_ (min^−1^)	0.00857 ± 0.00157	0.00987 ± 0.00067
t_1/2_ (min)	80.8 ± 14.8	70.2 ± 4.8

Data are expressed as the mean ± SD (*n* = 3). T_max_: time to reach peak whole concentration, C_max_: peak whole concentration, AUC: area under the concentration–time curve, AUMC: area under the moment of the concentration–time curve, MRT: mean residence time, CL: total body clearance, F: bioavailability, k_a_: absorption rate constant, k_el_: elimination rate constant, and t_1/2_: half-life time in elimination phase. ** *p* < 0.01 and * *p* < 0.05: statistically different from the RIS-OS/water-mixture group.

**Table 2:** Pharmacokinetic parameters of 9-OH-RIS following oral administration for the RIS-OS/water-mixture group and the RIS-OS/Dimbula tea-mixture group.

Parameter	RIS-OS/Water-Mixture Group	RIS-OS/Dimbula Tea-Mixture Group
T_max_ (min)	35 ± 23	80 ± 35
C_max_ (ng mL^−1^)	119.2 ± 5.4	74.7 ± 4.8 **
AUC (ng min mL^−1^)	15,100 ± 1400	16,223 ± 380
AUMC (ng min^2^ mL^−1^)	1.33 × 10^6^ ± 1.74 × 10^5^	2.2 × 10^6^ ± 3.1 × 10^5^ *
MRT (min)	87.5 ± 4.8	136 ± 16 *
CL/F (mL min kg^−1^)	21.6 ± 2.3	20.42 ± 0.82
k_el_ (min^−1^)	0.01096 ± 0.00033	0.01029 ± 0.00075
t_1/2_ (min)	63.2 ± 1.9	67.4 ± 4.9
Metabolic ratio	2.84 ± 0.29	3.14 ± 0.26

Data are expressed as the mean ± SD (*n* = 3). T_max_: time to reach peak whole concentration, C_max_: peak whole concentration, AUC: area under the concentration–time curve, AUMC: area under the moment of the concentration–time curve, MRT: mean residence time, CL: total body clearance, F: bioavailability, k_el_: elimination rate constant, and t_1/2_: half-life time in elimination phase. ** *p* < 0.01 and * *p* < 0.05: statistically different from the RIS-OS/water-mixture group.

**Table 3:** Pharmacokinetic parameters of the active moiety following oral administration for the RIS-OS/water-mixture group and RIS-OS/Dimbula tea-mixture group.

Parameter	RIS-OS/Water-Mixture Group	RIS-OS/Dimbula Tea-Mixture Group
T_max_ (min)	13.0 ± 2.9	65 ± 53
C_max_ (ng mL^−1^)	219 ± 23	102.5 ± 5.0 **
AUC (ng min mL^−1^)	20,430 ± 1258	21,293 ± 932

Data are expressed as the mean ± SD (*n* = 3). T_max_: time to reach peak whole concentration, C_max_: peak whole concentration, an AUC: area under the concentration–time curve. ** *p* < 0.01 statistically different from RIS-OS/water-mixture group.

## Data Availability

The original contributions presented in this study are included in the article. Further inquiries can be directed to the corresponding authors.

## Abbreviations

RIS-OS	Risperidone oral solution
RIS	Risperidone
IS	Internal standard
AUC_i.v.0–∞_	Area under the whole-blood concentration–time curve of intravenous
k_el_	Elimination rate constant
AUMC	Area under the (first) moment curve
MRT	Mean residence time
DBS	Dried blood spot

## References

[1] Leucht S., Tardy M., Komossa K., Heres S., Kissling W., Salanti G., Davis J.M. (2012). Antipsychotic drugs versus placebo for relapse prevention in schizophrenia: A systematic review and meta-analysis. Lancet.

[2] Keepers G.A., Laura M.D., Fochtmann J.M.D. (2020). The American Psychiatric Association Practice Guideline for the Treatment of Patients with Schizophrenia.

[3] Janssen Pharmaceuticals, Inc. (2025). Risperdal^®^ (Risperidone) Prescribing Information.

[4] Janssen Pharma K.K. (2021). RISPERDAL^®^ Oral Solution 1 mg/mL Drug Interview Form.

[5] Goromaru T., Fujita K., Mizumoto M., Ishizu T. (2023). Quantification of risperidone contained in precipitates produced by tea catechins using nuclear magnetic resonance. Chem. Pharm. Bull..

[6] Nishikawa Y., Suzuki H., Jinbayashi S., Sato N., Usui K., Okada K., Kawai S., Ohmukai K., Murai Y. (2025). Investigation of risperidone oral solution administration practices and risk of beverage-related drug interactions in Japanese patients. Drugs Ther. Perspect..

[7] International Tea Committee (2022). Annual Bulletin of Statistics.

[8] Leysen J.E., Janssen P.M., Megens A.A., Schotte A. (1994). Risperidone: A novel antipsychotic with balanced serotonin-dopamine antagonism, receptor occupancy profile, and pharmacologic activity. J. Clin. Psychiatry.

[9] Spina E., Avenoso A., Facciola G., Scordo M.G., Ancione M., Madia A., Ventimiglia A., Perucca E. (2000). Clinical pharmacokinetics of risperidone and 9-hydroxyrisperidone in relation to CYP2D6 metabolism. Clin. Pharmacokinet..

[10] Fukusumi K., Okamot Y. (2006). Evaluation of compatibility of risperidone with soft drinks and interactions of risperidone with tea tannin using isothermal titration microcalorimetry. Jpn. J. Pharm. Health Care Sci..

[11] Ikeda H., Moriwaki H., Yukawa M., Iwase Y., Aki H. (2010). Mechanism of interaction between risperidone and tea catechin (1) complex formation of risperidone with epigallocatechin gallate. Yakugaku Zasshi J. Pharm. Soc. Jpn..

[12] Ikeda H., Moriwaki H., Matsubara T., Yukawa M., Iwase Y., Yukawa E., Aki H. (2012). Mechanism of interaction between risperidone and tea catechin (2) influence of presence of galloyl group in catechin on insoluble complex formation with risperidone. Yakugaku Zasshi J. Pharm. Soc. Jpn..

[13] Jeganathan B., Müller A., Kuhnert N. (2018). Differentiation of black tea infusions according to origin, processing and botanical varieties using multivariate statistical analysis of LC-MS data. Food Res. Int..

[14] Zhang S., Long X., Xing Y., Ee K.H., Goh V.R.M., Huang Y., Pua A., Jublot L., Liu S.Q., Yu B. (2025). A systematic approach to analyzing catechins and catechin derivatives in Ceylon black tea using liquid chromatography coupled with triple quadrupole mass spectrometry. Food Chem. X.

[15] Goromaru T., Konishi N., Bunya M., Ishizu T. (2025). Solubilizing effect of caffeine on precipitate of risperidone oral solution and tea beverage. Biol. Pharm. Bull..

[16] Mannens G., Meuldermans W., Snoeck E., Heykants J. (1994). Plasma protein binding of risperidone and its distribution in blood. Psychopharmacology.

[17] Wagner J.G. (1993). Pharmacokinetics for the pharmaceutical. Scientist.

[18] Bondolfi G., Dufour H., Patris M., May J.P., Billeter U., Eap C.B., Baumann P. (1998). Risperidone versus clozapine in treatment-resistant chronic schizophrenia: A randomized double-blind study. Am. J. Psychiatry.

[19] Kondo A., Narumi K., Okuhara K., Takahashi Y., Furugen A., Kobayashi M., Iseki K. (2019). Black tea extract and theaflavin derivatives affect the pharmacokinetics of rosuvastatin by modulating organic anion transporting polypeptide (OATP) 2B1 activity. Biopharm. Drug Dispos..

